# Early Metabolic Profile in Neonates with Maternal Intrahepatic Cholestasis of Pregnancy

**DOI:** 10.3390/children12121655

**Published:** 2025-12-06

**Authors:** Bengisu Guner Yilmaz, Saygin Abali, Ariorad Moniri, Umut Kilinckaya, Ekin Altinbas, Beril Ay, Bengisu Karakose, Yusuf Sahinoglu, Melis Sahinoglu, Bugra Yilmaz, Mustafa Serteser, Ayse Korkmaz, Ozlem Pata, Serdar Beken

**Affiliations:** 1 Division of Neonatology, Department of Pediatrics, School of Medicine, Acibadem Mehmet Ali Aydinlar University, Istanbul 34638, Turkey; 2 Division of Pediatric Endocrinology, Department of Pediatrics, School of Medicine, Acibadem Mehmet Ali Aydinlar University, Istanbul 34638, Turkey; 3 School of Medicine, Acibadem Mehmet Ali Aydinlar University, Istanbul 34638, Turkey; 4 Department of Medical Biochemistry, School of Medicine, Acibadem Mehmet Ali Aydinlar University, Istanbul 34638, Turkey; 5 Division of Perinatology, Department of Obstetrics and Gynecology, School of Medicine, Acibadem Mehmet Ali Aydinlar University, Istanbul 34638, Turkey

**Keywords:** gestational intrahepatic cholestasis, amino acid profile, carnitine/acylcarnitine profile, metabolomics, neonate

## Abstract

**Highlights:**

**What are the main findings?**

- Neonates born to mothers with intrahepatic cholestasis of pregnancy (ICP) exhibit distinct perturbations in amino acid and acylcarnitine metabolism.
- Some metabolites emerged as strong individual discriminators, while a multimetabolite model demonstrated superior diagnostic accuracy (AUC = 0.86 ± 0.03) for identifying neonates affected by maternal ICP.

**What is the implication of the main finding?**

- Early metabolomic profiling may provide mechanistic insight into fetal responses to maternal cholestasis and may inform future approaches to neonatal risk assessment and follow-up.
- Findings may indicate mitochondrial β-oxidation and amino acid metabolism disruption as fetal adaptive responses to cholestasis.

**Abstract:**

**Background/Objectives**: Intrahepatic cholestasis of pregnancy (ICP) is associated with adverse perinatal outcomes. However, its metabolic consequences on newborns remain inadequately characterized. This study investigated amino acid, carnitine, and acylcarnitine profiles in neonates born to mothers with ICP. **Methods**: This retrospective study encompassed 299 neonates born to mothers with ICP. For comparative analysis, term infants without additional complications (ICP-term, *n* = 150) were compared with term controls (*n* = 150). Capillary blood samples collected at 24–48 h of life as part of newborn screening were analyzed using LC–MS/MS for acylcarnitine and amino acid profiles. **Results**: The ICP cohort exhibited a high preterm delivery rate (46.2%), with maternal bile acids negatively correlating with gestational age (r = −0.266, *p* < 0.001). No inborn errors of metabolism were observed. Elevated levels of amino acids (alanine, leucine/isoleucine, valine, tyrosine, arginine, glycine, and ornithine) and specific acylcarnitines (C5, C5-OH, C10:1, and C18:2), along with decreased levels of amino acids (argininosuccinic acid and glutamic acid) and specific acylcarnitines (C3, C5-DC, C6-DC, C14, C14:1, C16, C16:1, and C18:1-OH), were observed in ICP-term neonates (*p* < 0.05). Receiver operating characteristic curve analysis identified ornithine (area under the curve [AUC] = 0.74) and leucine/isoleucine (AUC = 0.73) as strong discriminators. A multivariable model integrating multiple metabolites achieved high accuracy (AUC = 0.86 ± 0.03). **Conclusions**: This first comprehensive characterization of neonatal metabolic alterations in ICP reveals amino acid metabolism, fatty acid oxidation, and mitochondrial function disruptions, suggesting fetal adaptation to a cholestatic intrauterine environment. Metabolomic profiling may improve understanding of maternal–fetal interactions and inform strategies for risk stratification and long-term monitoring.

## 1. Introduction

Intrahepatic cholestasis of pregnancy (ICP) is the most common pregnancy-specific liver disorder, with a global incidence of 0.1–4%. It frequently presents in the second or third trimester with otherwise unexplained pruritus and elevated maternal total bile acid (TBA) levels, typically normalizing within 48 h postpartum. Multiple gestations, advanced maternal age, and hepatitis C infection are established risk factors [1,2]. Although the precise pathophysiological mechanisms remain incompletely understood, cholestatic effects of reproductive hormones, particularly 17β-estradiol and sulfated progesterone metabolites, in genetically susceptible females potentially impair hepatobiliary transport and alter bile acid composition, causing increased circulating TBA levels. An elevated TBA level is the most sensitive and occasionally the only biochemical abnormality; however, alanine aminotransferase, aspartate aminotransferase, γ-glutamyl transferase, and serum lipid levels may also be elevated [2,3]. Beyond maternal morbidity, ICP is associated with adverse perinatal outcomes, including spontaneous preterm birth, fetal distress, and stillbirth. Intrauterine metabolic milieu alterations during ICP may also affect fetal intermediary metabolism; however, studies on this aspect are scarce.

Carnitine, acylcarnitine, and amino acid measurements in dried blood spots obtained immediately following birth form the cornerstone of newborn screening (NS) for inborn errors of metabolism. These analytes reflect molecular and phenotypic alterations in intermediate metabolites, facilitating prompt detection of heritable biochemical disturbances [4]. Along with diagnosing primary metabolic disorders, NS metabolite profiles have been employed for detecting secondary metabolic perturbations associated with non-genetic maternal or perinatal conditions, including gestational diabetes mellitus and preeclampsia [5,6,7,8,9,10].

Through hepatotoxic lipid metabolite accumulation, fetal fatty acid oxidation defects (FAODs) may contribute to pregnancy-related liver disorders; however, their potential association with ICP remains largely unexplored [11,12,13]. The low prevalence of FAODs and ICP poses challenges to establishing this association.

The objectives of this study were to:

(i) Assess the amino acid, carnitine, and acylcarnitine profiles of neonates born to mothers with ICP.

(ii) Compare these metabolic profiles with those of healthy term neonates to identify potential metabolic differences.

(iii) Provide novel insights into the possible metabolic consequences of maternal ICP on the offspring.

## 2. Materials and Methods

### 2.1. Study Population and Design

This retrospective study was conducted at a tertiary care center and encompassed neonates born to mothers diagnosed with ICP from January 2016 to December 2021. The institutional ethics committee approved the study protocol (ATADEK 2021-14/33).

The ICP cohort comprised all neonates of any gestational age born to mothers with ICP, defined as maternal serum TBA levels ≥ 10 µmol/L. For subgroup analyses, maternal TBA levels were stratified into the following three categories: 10–39, 40–99, and ≥100 μmol/L [14]. Demographic and clinical characteristics, as well as amino acid, carnitine, and acylcarnitine profiles obtained via tandem mass spectrometry (LC–MS/MS), were recorded. Multiple pregnancies were included, while mothers with co-existing conditions such as gestational diabetes mellitus (GDM), hypertension, and type 1 diabetes were excluded. From this cohort, term infants (gestational age ≥ 37 weeks) without additional perinatal complications like hypoxic–ischemic encephalopathy, intracranial hemorrhage, early neonatal sepsis, hypoglycemia or congenital anomalies were selected as the ICP-term group. These infants were compared with a control group comprising healthy term neonates without additional perinatal complications or congenital anomalies and who were born at the same institution during a similar time period. The control group consisted of neonates matched to the ICP-term group for birth weight (BW) standard deviation scores (SDSs) for gestational week (GW), born to mothers without any gestational or other medical conditions. Birth weight SDSs for GW were calculated on the basis of national reference standards provided by the Child Metrics system [15,16]. For all study groups, demographic and clinical variables, including sex, gestational age, BW and metabolic screening results, were obtained from electronic medical records. Capillary blood samples collected at 24–48 h of life as part of newborn screening and stored in sealed plastic bags at 2–8 °C until transported to the laboratory.

### 2.2. LC–MS/MS Analysis

Dried blood spot samples were punched into 3.2 mm diameter disks from filter paper [Schleicher and Schuell (S&S) no. 903, Schleicher & Schuell, Inc., Keene, NH, USA] and placed into 96-well polypropylene microplates. A total of 200 µL of standard solution containing methanol and a mixture of isotope-labeled internal standards (IS) for amino acids and acylcarnitines was added to each well. Isotope-labeled standards for amino acids (Set A, Cat. No: NSK-A-1) and acylcarnitines (Set B, Cat. No: NSK-B-1) were obtained from Cambridge Isotope Laboratories (Tewksbury, MA, USA). Sample extraction was performed using a rotary shaker at room temperature for 30 min. The extracted supernatant was transferred to a new plate, and 60 µL of butanolic HCl was added to each well for derivatization, followed by incubation at 65 °C for 25 min. Subsequently, the samples were evaporated at 45 °C for 1 h. Finally, 100 µL of mobile phase (acetonitrile:water, 80:20 *v*/*v*) was added, and the plate was shaken for 10 min prior to analysis.

Dried blood spot quality control materials for amino acids and acylcarnitines were obtained from the Centers for Disease Control and Prevention [CDC, Atlanta, GA, USA, Newborn Screening Quality Assurance Program (NSQAP)]. The Newborn Screening Quality Assurance Program of the CDC (Atlanta, GA, USA) supplied the quality control materials for DBS analysis of amino acids and acylcarnitines. Quantitative analysis was performed using flow injection analysis–tandem mass spectrometry (FIA–MS/MS) on a Shimadzu LCMS-8040 Triple Quadrupole Mass Spectrometer (Shimadzu Corporation, Kyoto, Japan), a method that enables rapid direct detection of analytes without chromatographic separation. Quantification was achieved by calculating the signal intensity ratio of each analyte to its corresponding internal standard. Data acquisition and processing were performed in multiple reaction monitoring mode using Shimadzu’s LabSolutions LCMS software (Version 5.60 SP2). Amino acid and acylcarnitine final concentrations were determined using the Neonatal Mass Screening Software (version 2.20), a component of the Neonatal Solution platform tailored for NS applications.

### 2.3. Statistical Analysis

Statistical analysis was performed using the Statistical Package for the Social Sciences (SPSS, version 16.0; SPSS Inc., Chicago, IL, USA). Continuous variables were presented as medians and interquartile ranges, and categorical variables were expressed as frequencies and percentages. The normality of distribution for continuous variables was evaluated using the Kolmogorov–Smirnov test in conjunction with graphical methods, including histograms and Q–Q plots. For group comparisons, Student’s *t*-test was applied to normally distributed continuous variables, whereas the Mann–Whitney U test was employed for non-normally distributed variables. Comparisons of categorical variables between groups were performed using the Pearson chi-square test or Fisher–Freeman–Halton test, as appropriate. Correlation analyses between continuous variables were performed using Pearson’s correlation coefficient and Spearman’s rank correlation for linear and monotonic associations, respectively. Moreover, to assess underlying metabolic patterns, multivariate statistical techniques were employed. To identify key contributors to the variance in metabolic profiles, principal component analysis (PCA) was conducted. The PCA was used to explore overall data structure and identify variables contributing most strongly to variance, rather than to infer statistical group differences. Receiver operating characteristic (ROC) curve analyses were performed to evaluate the diagnostic performance of individual and combined metabolites. Univariable and multivariable logistic regression models were employed to assess the predictive value of metabolic markers. Cross-validation techniques, including 10-repetition, fivefold cross-validation (totaling 50 model runs), were applied to minimize overfitting and estimate the generalizability of the classification models.

## 3. Results

In the ICP cohort of 299 neonates (148 females) born to mothers with ICP, the preterm delivery rate was 46.2%, with a mean GW at birth of 36.3 ± 0.1 weeks (median: 37.0). The median BW was 2913 g, and 1.3% of infants were classified as small for gestational age (SGA). Neonatal intensive care unit admission was necessitated for 20.7% of neonates. Maternal serum biochemistry revealed a mean TBA level of 34.5 (range, 10.0–186.6) μmol/L. The clinical characteristics of the neonates and the associated maternal biochemical profile are presented in Table 1.

In the ICP cohort, elevated metabolite levels were detected for tyrosine in one patient (360.13 μmol/L), valine in three patients (299.6–304.8 μmol/L), ornithine in one patient (358.84 μmol/L), C3 in one patient (6.92 μmol/L), and C14:1 in one patient (0.76 μmol/L). Elevated C6 levels (0.30–1.77 μmol/L), detected in 23 patients, was the most frequently observed abnormality. None of these abnormalities were considered clinically significant, and no inborn metabolic errors were identified in the cohort. The metabolite profiles of the ICP-term group are shown in Table A1.

**Figure 1:**
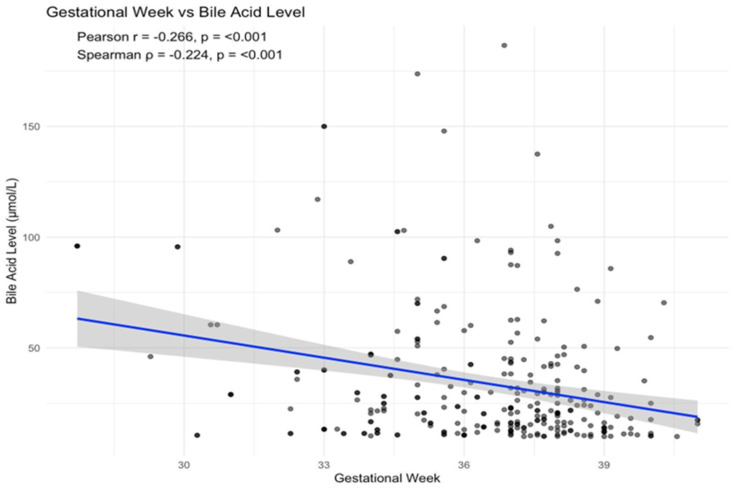
Correlation between serum bile acid levels and gestational week.

Gestational age was significantly negatively correlated with TBA levels (Pearson’s r = −0.266, *p* < 0.001; Spearman’s ρ = −0.224, *p* < 0.001), indicating a gradual decline in TBA levels with advancing gestation (Figure 1). Preterm delivery rates significantly differed across maternal TBA categories (10–39 µmol/L, 41.7%; 40–99 µmol/L, 54.4%; and ≥100 µmol/L, 83.3%; χ^2^ = 10.13, df = 2, *p* = 0.006).

### Comparison of Amino Acid and Carnitine/Acylcarnitine Profiles Between the ICP-Term and Control Groups

**Figure 2:**
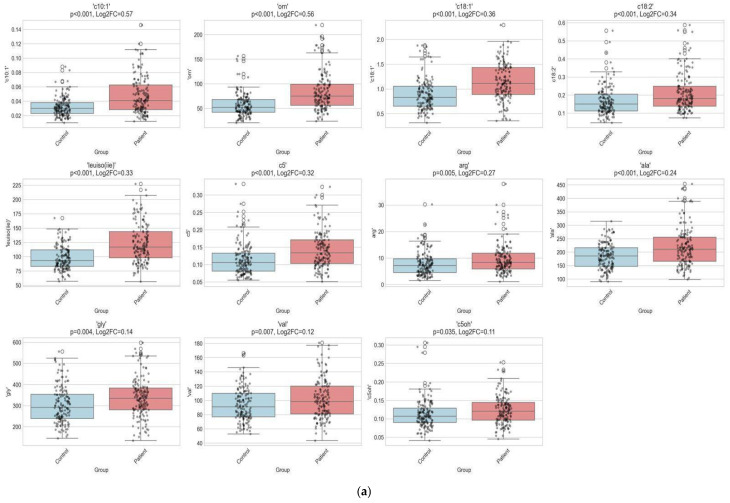

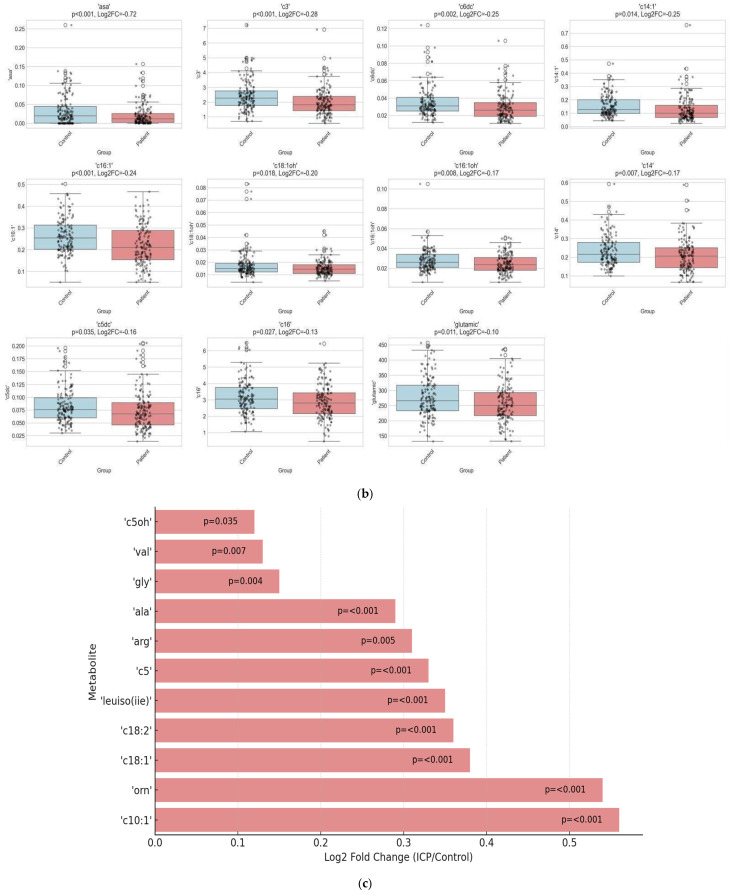

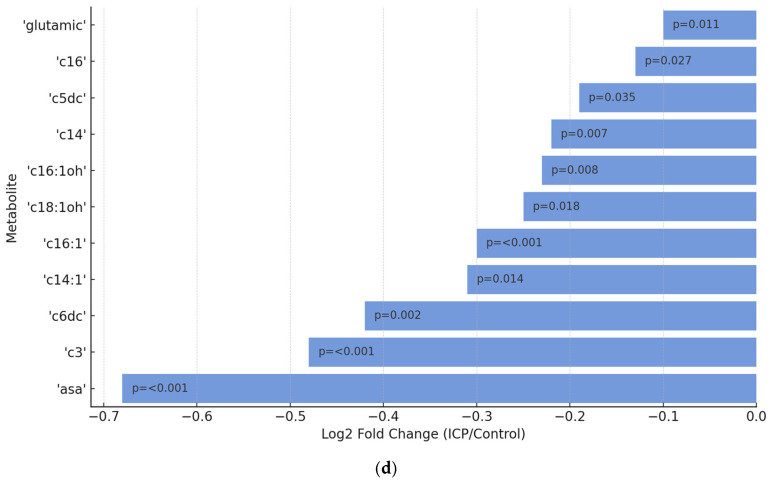
Metabolomic alterations in neonates born to mothers with intrahepatic cholestasis of pregnancy. (**a**) **Boxplots of the top increased metabolites in cholestasis:** this panel presents boxplots comparing the top 12 significantly increased metabolites in the ICP-term group versus controls. (**b**) **Boxplots of the top decreased metabolites in cholestasis:** this panel displays the top 12 significantly decreased metabolites in the ICP-term group. (**c**) **Barplot of the top increased metabolites (log2 fold change [FC]):** this horizontal barplot visualizes the log2FC of significantly increased metabolites in the ICP group. (**d**) **Barplot of the top decreased metabolites (log2FC):** this panel illustrates the most decreased metabolites in the cholestasis group.

*The comparison of amino acid and carnitine/acylcarnitine profiles* is presented in Table A2. Both groups encompassed 150 neonates with >37 GW each and demonstrated no significant difference in terms of BW SDS (*p* > 0.05). A distinct panel of metabolites was significantly increased and decreased in neonates born to mothers with ICP (Figure 2). The ICP-term group exhibited higher levels of alanine, leucine/isoleucine, valine, tyrosine, arginine, glycine, ornithine, C5, C5-OH, C10:1, C18:1, C18:2, and C18:2-OH than controls (*p* < 0.05). Among the most prominently increased metabolites were C10:1 and ornithine, both showing Log2 fold changes approaching +0.5 with *p*-values of <0.001, indicating highly significant upregulation. Furthermore, C18:1, C18:2, and C5 levels demonstrated marked elevations. The ICP-term group showed significantly decreased arginosuccinic acid, glutamic acid, C3, C5-DC, C6-DC, C12, C14, C14:1, C16, C16:1, C16:1-OH, and C18:1-OH levels (*p* < 0.05). Among these markers, arginosuccinic acid, C3, and C6-DC demonstrated the greatest decreases, with log2 fold changes of −0.72, −0.45, and −0.40, respectively, all with *p*-values of <0.01, indicating strong statistical significance. Several long-chain acylcarnitines, including C14:1, C16:1, and C18:1-OH, also exhibited moderate but consistent reductions.

**Figure 3:**
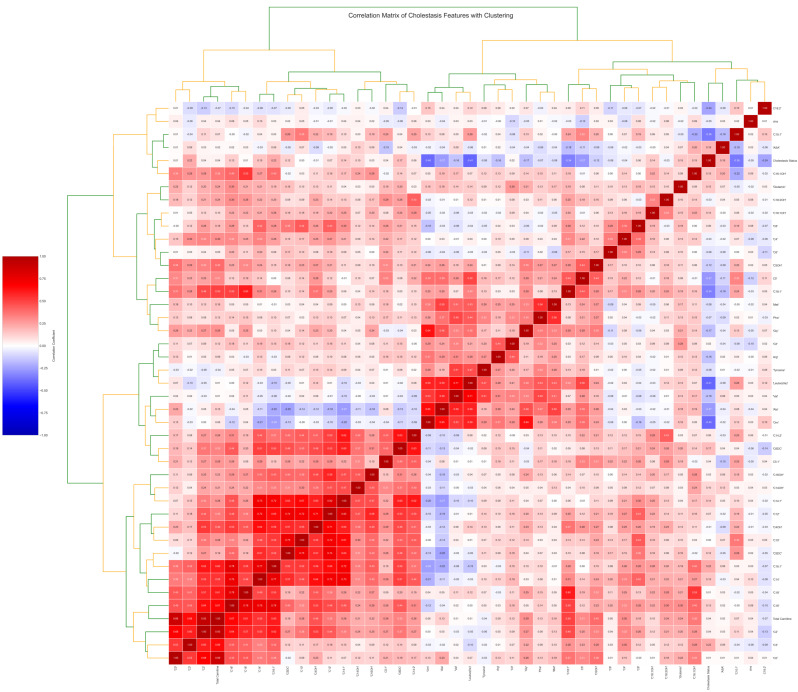
**Heatmap of correlations between metabolomic profiles and cholestasis diagnosis with clustering.** The correlation heatmap with hierarchical clustering illustrates the complex interrelationships among clinical and metabolic variables in neonates born to mothers with ICP. Each cell represents the Pearson correlation coefficient between pairs of variables, with red and blue shades indicating positive and negative correlations, respectively.

Prominent clusters of strongly correlated metabolites, particularly among medium- and long-chain acylcarnitines, as well as among specific amino acids, suggest strong statistical relationships. These observed clusters are consistent with the known biochemical origins of these metabolites within related metabolic pathways (Figure 3). Notably, C10:1, ornithine, and leucine/isoleucine, which were significantly increased in the cholestasis group, were positively correlated with each other. Conversely, metabolites that were decreased, including arginosuccinic acid, C3, and C6-DC, formed a distinct negatively correlated cluster.

**Figure 4:**
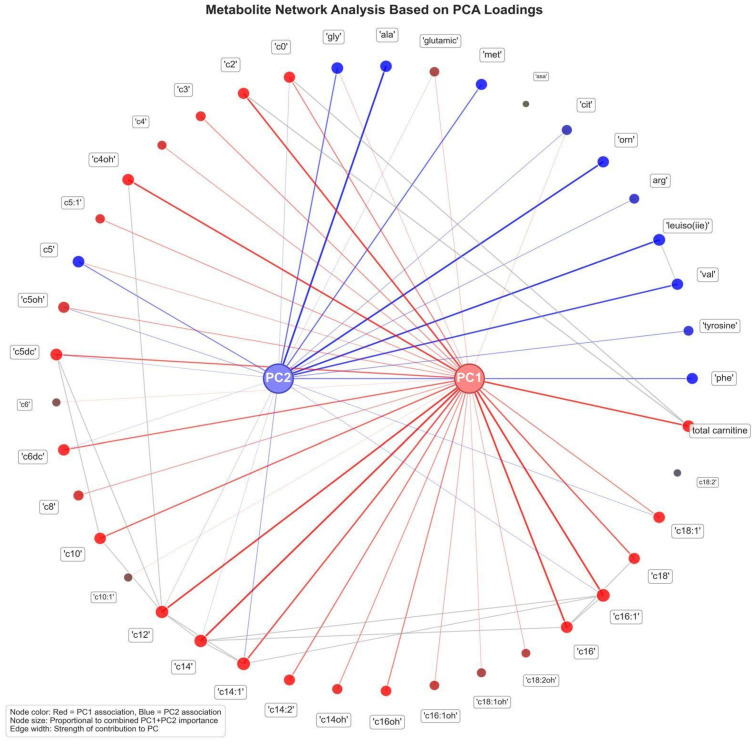
**Metabolite network analysis based on PCA loadings.** Each node denotes an individual metabolite, with node size proportional to its overall contribution to the PCA model and color indicating its primary association with either the first principal component (PC1, red) or the second principal component (PC2, blue). Edges connect metabolites to the principal components, with line thickness reflecting the strength of each metabolite’s contribution. Long-chain acylcarnitines, including c14, c16, c18, c18:1, and total carnitine, mainly trigger PC1, demonstrating that lipid metabolism and fatty acid oxidation are major sources of variation between the ICP-term and control groups. Amino acids and short-chain acylcarnitines, including valine, phenylalanine, tyrosine, leucine, and glycine, as well as c2, c3, and c4, primarily influence PC2, indicating amino acid metabolism alterations.

A metabolite network analysis based on PCA loadings, underscoring the major metabolic contributors to the variance observed between neonates born to mothers with ICP-term and controls, is illustrated in Figure 4. PC1 was predominantly characterized by elevated C16, C18, C18:1, C14:1, and total carnitine levels, suggesting a shift in lipid metabolism and carnitine-dependent mitochondrial β-oxidation in the ICP-term group. In contrast, PC2 was mainly characterized by amino acids and short-chain acylcarnitines, including ornithine, valine, leucine/isoleucine, arginine, glycine, and alanine, reflecting amino acid catabolism and nitrogen handling alterations. The dual contribution of metabolites, such as glutamic acid, glycine, and C0, to PC1 and PC2 suggests involvement in multiple intersecting pathways.

**Figure 5:**
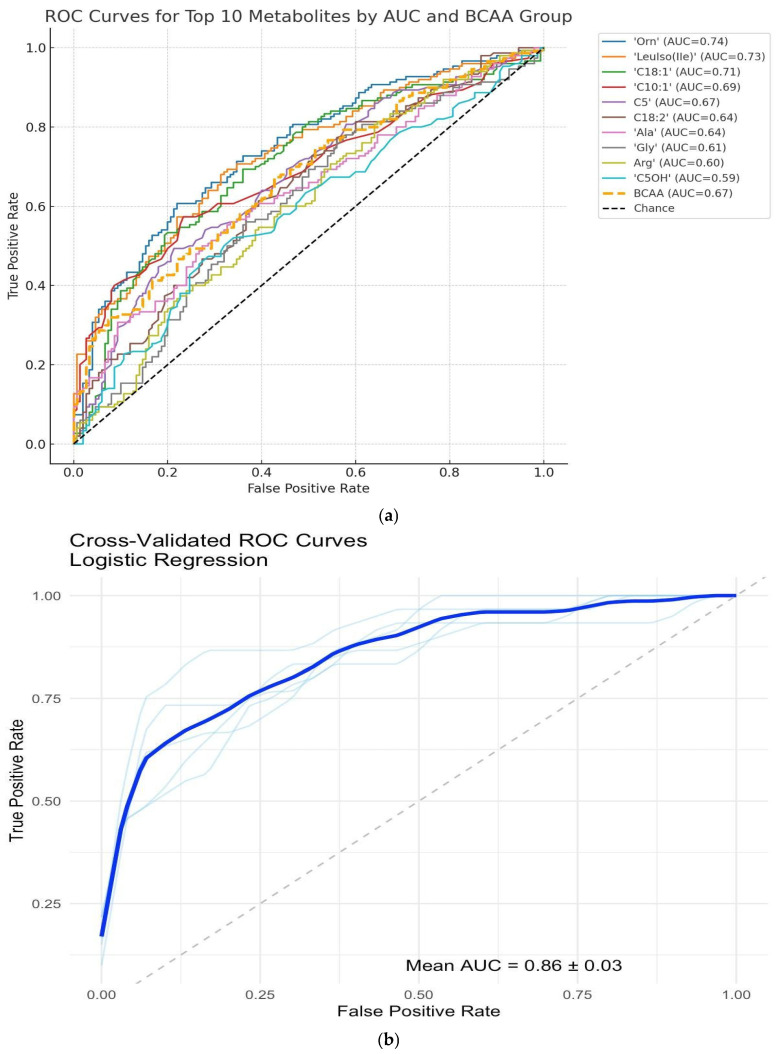
**Diagnostic Performance of Individual and Multivariable Metabolite Models for ICP.** (**a**) **Receiver operating characteristic** (**ROC) curves for the top 10 individual metabolites ranked by area under the curve (AUC).** Each curve represents the diagnostic performance of a logistic regression model trained on a single metabolite for differentiating neonates born to mothers with ICP from controls. (**b**) **Cross-validated ROC curve of the multivariable logistic regression model using all quantified metabolites.** The plot illustrates the ROC curves derived from fivefold cross-validation of a logistic regression model trained on the full metabolite panel for distinguishing neonates born to mothers with ICP from controls. The mean AUC is 0.86 ± 0.03, indicating discriminative performance. Light blue lines denote individual fold ROC curves, whereas the dark blue line indicates the mean ROC. The model’s performance reflects the additive diagnostic value of several metabolites.

The ROC curves for the top 10 individual metabolites ranked by their area under the curve (AUC), providing insight into the diagnostic utility of single biomarkers for distinguishing neonates born to mothers with ICP from healthy controls, are depicted in Figure 5a. Among these biomarkers, ornithine demonstrated the highest classification performance (AUC = 0.74), closely followed by leucine/isoleucine (AUC = 0.73) and C18:1 (AUC = 0.71). Additional contributors, including C10:1 (AUC = 0.69), C5 (AUC = 0.67), and C18:2 (AUC = 0.64), exhibited moderate predictive accuracy. Amino acids, including alanine, glycine, and arginine, yielded lower AUC values (0.64–0.60), whereas C5OH demonstrated the weakest performance (AUC = 0.59). The ROC curves generated from a multivariable logistic regression model incorporating the complete panel of quantified metabolites to differentiate between neonates born to mothers with ICP and healthy controls are presented in Figure 5b. An analysis of the coefficients from the full model revealed several key metabolites that contributed most strongly to the predictive model. Among the most influential were ornithine (coefficient: +2.10) and C18:1 (coefficient: +1.82), both of which were elevated in the ICP group and positively associated with disease classification. Conversely, C3 and C18:1OH had negative coefficients (−1.14 and −1.13, respectively), indicating reduced levels in cholestasis and inversely related to ICP diagnosis (AUC = 0.86 ± 0.03). This high AUC value shows that the combined metabolic profile yields strong diagnostic accuracy, significantly outperforming any single metabolite in isolation. Notably, the model’s consistency across folds reflects robust generalizability and limited overfitting. Collectively, this model demonstrates the power of integrated metabolite profiling for the accurate and biologically meaningful classification of disease states.

## 4. Discussion

ICP is associated with impaired placental function, which may lead to suboptimal fetal nutrient delivery and an increased risk of fetal growth restriction. In our study, the ICP cohort demonstrated a higher incidence of preterm birth. Consistent with previous reports, our findings also demonstrated that higher maternal bile acid levels were associated with lower gestational age and an increased rate of preterm birth [17,18]. Several studies have demonstrated that early postnatal metabolic profiles significantly differ between SGA and large-for-gestational-age (LGA) infants, reflecting differences in intrauterine growth patterns and nutrient availability, regardless of the underlying maternal etiology [19]. Furthermore, gestational age has been demonstrated to influence neonatal metabolic profiles, with distinct variations in amino acid and acylcarnitine levels [20]. These findings highlight the significance of considering fetal growth status and maturity when interpreting metabolomic data. Therefore, premature infants were excluded from comparisons with the control group and ensuring that BW SDS did not significantly vary between the groups received particular attention.

A limited number of studies have investigated neonatal metabolite profiles to identify metabolic signatures associated with pregnancy complications affecting the newborn. Reiss et al. demonstrated metabolic evidence of insulin resistance in newborns of diabetic mothers, characterized by elevated short-chain acylcarnitines and free carnitine. Similarly, increased acylcarnitine and free carnitine levels were reported in neonates born to hypertensive and pre-eclamptic mothers [9,10]. These findings collectively suggest that maternal metabolic disturbances can alter neonatal acylcarnitine patterns. In line with this, our study demonstrated distinct alterations in carnitine and acylcarnitine profiles in neonates born to mothers with ICP, supporting the concept that maternal metabolic or hepatic dysfunction may influence neonatal energy metabolism. These ICP-related alterations appear to reflect functional and adaptive metabolic responses rather than pathological blocks, as none of the infants fulfilled biochemical or clinical criteria for an inborn error of metabolism. This supports the interpretation that the observed metabolite changes arise from transient intrauterine exposure to maternal cholestasis, rather than underlying neonatal metabolic disease.

Carnitines and acylcarnitines are significantly involved in FAO. Recent advances have expanded our understanding of its involvement beyond FAO, emphasizing its contribution in diverse biological processes [21]. Carnitines and acylcarnitines serve as metabolic intermediates and signaling molecules that are actively exchanged between tissues. This dynamic exchange influences the regulation of several metabolic and physiological pathways across various organ systems, highlighting the critical role of carnitines and acylcarnitines in metabolic integration. Carnitines and acylcarnitines modulate insulin sensitivity within the endocrine system through direct and indirect mechanisms, and a consensus exists that heightened long-chain acylcarnitine levels are associated with insulin resistance, and their buildup is increasingly recognized as a possible biomarker for impaired insulin sensitivity [22]. In our study, the groups demonstrated no significant difference in terms of overall long-chain acylcarnitine levels; however, analysis of individual long-chain fatty acids revealed distinct alterations. Examination of individual acylcarnitines, rather than grouping them into short-, medium-, and long-chain species, revealed distinct and heterogeneous alterations, suggesting that metabolic changes did not occur uniformly across chain-length categories. These findings underscore the importance of evaluating individual acylcarnitine species rather than aggregated groups to more accurately characterize the specific metabolic perturbations associated with ICP. Giacco et al. investigated how the saturation level of fatty acids and their corresponding acylcarnitines influences the direct effects of active thyroid hormones and insulin sensitivity in skeletal muscle cells. They observed that saturated fatty acid, palmitate (C16), tends to induce insulin resistance, whereas unsaturated fatty acids, oleate (C18:1) and linoleate (C18:2), improve insulin sensitivity [23]. As demonstrated in our study, C16 levels were reduced, whereas C18:1 and C18:2 levels were elevated in the ICP-term group; this pattern may reflect a metabolic adaptation aimed at enhancing insulin sensitivity. Moreover, Carland et al. revealed the inhibitory effects of C18:1 on glycine transporter 2 (GlyT2). GlyT2 is significantly involved in regulating glycine levels in the central nervous system, particularly within inhibitory glycinergic neurons, and holds promise as an analgesic [24].

In our study, elevated alanine, glycine, ornithine, and tyrosine levels were observed, consistent with the findings in SGA infants [19,20]. The increase in alanine and glycine levels suggests an altered redox state arising from impaired mitochondrial respiratory chain function [25]. Additionally, alanine is significantly involved in lactate metabolism and facilitates glucose homeostasis. Elevated alanine levels have been identified as major predictors of altered metabolic pathways and may lead to a higher risk of developing metabolic syndrome-related conditions later in life [26].

Several studies have identified branched-chain amino acids (BCAAs) and their catabolic derivatives, including C3 and C5 acylcarnitines, as potential insulin resistance biomarkers [27]. Impaired mitochondrial function in individuals with obesity or diabetes is linked to a marked increase in circulating BCAA levels [28]. Conversely, improvements in mitochondrial function have been demonstrated to prevent such increases, indicating a close interplay between mitochondrial efficiency and BCAA metabolism in metabolic disorders. Leucine, an essential BCAA, plays a crucial role in protein and energy metabolism regulation. Leucine enhances energy metabolism by promoting glucose uptake, stimulating mitochondrial biogenesis, and increasing fatty acid oxidation, thereby supporting the cellular energy demands required for growth and metabolic homeostasis [29,30]. In our study, neonates born to mothers with ICP showed increased leucine/isoleucine, isovalerylcarnitine (C5), and 3-hydroxyisovalerylcarnitine (C5OH) levels, as well as reduced glutarylcarnitine (C5DC) levels. In the catabolic pathway of leucine, isovaleryl-CoA and 3-hydroxyisovaleryl-CoA are produced through a series of mitochondrial enzymatic reactions. These intermediate acyl-CoA compounds are subsequently conjugated with carnitine to facilitate their transport out of the mitochondria. Specifically, isovaleryl-CoA generates C5, whereas 3-hydroxyisovaleryl-CoA produces C5OH. The concentrations of these acylcarnitine derivatives reflect the integrity and efficiency of leucine metabolism. C5DC functions as a key marker for lysine and tryptophan catabolism. Lysine and tryptophan degradation causes glutaryl-CoA formation. This metabolite accumulates in the presence of a glutaryl-CoA dehydrogenase deficiency, prompting its conjugation with carnitine to form C5DC. Consequently, elevated C5DC levels indicate impaired lysine and tryptophan metabolism, raising suspicion for underlying metabolic disorders, including glutaric acidemia type 1 [31].

The metabolite network analysis based on PCA loadings underscore the major metabolic contributors to the variance observed between neonates born to mothers with ICP and controls. Our findings demonstrate a shift in lipid metabolism and carnitine-dependent mitochondrial β-oxidation and amino acid catabolism and nitrogen handling alterations. This integrative network reveals that the metabolic disturbances in ICP are not isolated but rather organized into coordinated clusters showing key shifts in energy metabolism, amino acid turnover, and mitochondrial function, thereby offering insights into fetal adaptive responses to the cholestatic intrauterine environment.

This study has several limitations. First, it was conducted retrospectively, which may restrict the ability to infer causality. Second, the available clinical data were limited to information extracted from medical records. Third, there is no standardized treatment protocol for intrahepatic cholestasis of pregnancy, which may have led to variability in maternal management and, consequently, neonatal metabolic profiles. Further prospective studies with standardized clinical follow-up are needed to confirm and expand these findings.

## 5. Conclusions

This study provides valuable insights into the metabolic profiles of neonates born to mothers with ICP. The findings suggest that clinicians interpreting newborn screening results should consider maternal ICP as a potential factor influencing neonatal metabolite profiles. These early-life changes, detectable through neonatal metabolomic profiling, may have long-term implications for metabolic health and support the concept of fetal origins of adult-onset diseases.

## Figures and Tables

**Table 1:** Clinical characteristics of neonates born to mothers with ICP and associated maternal biochemical profile.

**Gestational age, *week*, mean ± SD (range)**	**36.3 ± 0.1 (27.7–41.0)**
**Preterm**, *n* (%)	138 (46.2)
**Female**, *n* (%)	148 (49.5)
**Maternal age**, *year*, mean ± SD (range)	33.1 ± 0.3 (30.0–44.6)
**Birth weight**, *g*, median (range)	2913 (750–4180)
**LBW**, *n* (%)	83 (27.8)
**VLBW**, *n* (%)	6 (2.0)
**ELBW**, *n* (%)	2 (0.7)
**Birth weight SDS**, mean ± SD (range)	0.4 ± 0.1
**SGA**, *n* (%)	4 (1.3)
**NICU admission**, *n* (%)	62 (20.7)
***Maternal serum biochemical profile*** *(mean ± SD, range)*	
**Bile acid**, *µmol/L* (*n* = 299)	34.5 ± 1.7 (10.0–186.6)
**ALT**, *IU/L* (*n* = 281)	151.3 ± 11.9 (9–1562)
**AST**, *IU/L* (*n* = 277)	76.0 ± 5.1 (10–815)
**GGT**, *IU/L* (*n* = 155)	32.7 ± 2.2 (6–179)
**ALP**, *IU/L* (*n* = 153)	195.8 ± 6.6 (23–608)
**Total bilirubin**, *mg/dL* (*n* = 162)	0.54 ± 0.03 (0.2–2.2)
**Direct bilirubin**, *mg/dL* (*n* = 148)	0.29 ± 0.02 (0.1–2.1)

ICP, intrahepatic cholestasis of pregnancy; SDS, standard deviation score; SGA, small for gestational age; LBW, low birth weight; VLBW, very low birth weight; ELBW, extremely low birth weight; NICU, neonatal intensive care unit; ALT, alanine transaminase; AST, aspartate transaminase; GGT, gamma-glutamyl transferase; ALP, alkaline phosphatase.

## Data Availability

The raw data supporting the conclusions of this article will be made available by the authors on request.

## Abbreviations

The following abbreviations are used in this manuscript:

ICP	Intrahepatic cholestasis of pregnancy
LC–MS/MS	Liquid chromatography–mass spectrometry/mass spectrometry
AUC	Area under the curve
TBA	Total bile acid
NS	Newborn screening
FAODs	Fatty acid oxidation defects
BW	Birth weight
SDSs	Standard deviation scores
GW	Gestational week
FIA–MS/MS	Flow injection analysis–tandem mass spectrometry
IQR	Interquartile range
PCA	Principal component analysis
ROC	Receiver operating characteristic
SGA	Small for gestational age
LBW	Low birth weight
VLBW	Very low birth weight
ELBW	Extremely low birth weight
NICU	Neonatal intensive care unit
ALT	Alanine transaminase
AST	Aspartate transaminase
GGT	Gamma-glutamyl transferase
ALP	Alkaline phosphatase
PC	Principal component
GlyT2	Glycine transporter 2
BCAAs	Branched-chain amino acids

## Appendix A

**Table A1:** Amino acid, carnitine, and acylcarnitine levels in dried blood spots of neonates born to mothers with intrahepatic cholestasis of pregnancy.

Amino Acid, Units (Reference Values)	Median (IQR)
Alanine, μmol/L (90–900)	215.39 (98.47–523.92)
Methionine, μmol/L (9.0–65),	20.42 (10.63–45.09)
Phenylalanine, μmol/L (22.9–120)	49.46 (30.58–92.49)
Tyrosine, μmol/L (26.9–275)	87.52 (17.68–360.13)
Leucine/Isoleucine (IIe), μmol/L (43.4–373)	122.76 (56.38–237.07)
Valine, μmol/L (52.8–250)	101.90 (28.90–306.87)
Arginine, μmol/L (0.0–50)	8.75 (1.14–46.47)
Citrulline, μmol/L (2.0–65)	10.43 (3.45–37.07)
Glycine, μmol/L (105–1100)	336.67 (134.57–597.55)
Ornithine, μmol/L (19.7–300)	76.64 (20.02–358.84)
Arginino-succinic acid, μmol/L (0.0–0.66)	0.015 (0.00–0.25)
Glutamic acid, μmol/L (0.0–704)	260.07 (132.32–531.08)
**Acylcarnitines, units (reference values)**	
Free carnitine (C0), μmol/L (8.6–90.0)	22.75 (10.36–56.27)
Total carnitine, μmol/L	53.08 (23.22–131.50)
**Short-chain acylcarnitines**	
Acetyl carnitine (C2), μmol/L (5.0–73.4)	20.75 (5.98–64.31)
Propionyl carnitine (C3), μmol/L (0.0–6.8)	1.72 (0.26–6.92)
Butyryl carnitine (C4), μmol/L (0.0–1.2)	0.25 (0.06–1.08)
Isovaleryl carnitine (C5), μmol/L (0.0–0.6)	0.01 (0.00–0.08)
Tiglyl carnitine (C5:1), μmol/L (0.0–0.13)	0.15 (0.05–0.58)
**Medium-chain acylcarnitines**	
Hexonyl carnitine (C6), μmol/L (0.0–0.21)	0.05 (0.01–1.77)
Octanoyl carnitine (C8), μmol/L (0.0–0.32)	0.05 (0.00–0.30)
Decanoyl carnitine (C10), μmol/L (0.0–0.48)	0.06 (0.02–0.25)
Decenoyl carnitine (C10:1), μmol/L (0.0–0.28)	0.04 (0.01–0.19)
Dodecanoyl carnitine (C12), μmol/L (0.0–0.69)	0.08 (0.03–0.40)
**Long-chain acylcarnitines**	
Tetradecanoyl carnitine (C14), μmol/L (0.0–0.8)	0.18 (0.05–0.77)
Tetradecenoyl carnitine (C14:1), μmol/L (0.0–0.6)	0.08 (0.02–0.76)
Tetradecadienoyl carnitine (C14:2), μmol/L (0.0–0.25)	0.02 (0.00–0.11)
Palmitoyl carnitine (C16), μmol/L (0.0–8.70)	2.40 (0.39–6.44)
Palmitoleyl carnitine (C16:1), μmol/L (0.0–1.04)	0.18 (0.03–0.51)
Stearyl carnitine (C18), μmol/L (0.0–2.24)	0.79 (0.20–1:98)
Oleyl carnitine (C18:1), μmol/L (0.0–2.8)	1.11 (0.26–2.55)
Linolenoyl carnitine (C18:2), μmol/L (0.0–0.9)	0.20 (0.07–0.68)
**Dicarboxylic acids-DC-derived acylcarnitine esters**	
Glutaryl carnitine (C5-DC), μmol/L (0.0–0.21)	0.06 (0.01–0.21)
Methylglutaryl carnitine (C6-DC), μmol/L (0.0–0.20)	0.02 (0.01–0.11)
**Hydroxylated acids-OH-derived acylcarnitine esters**	
3-OH butyryl carnitine (C4-OH), μmol/L (0.0–0.48)	0.13 (0.04–0.48)
3-OH isovaleryl carnitine (C5-OH), μmol/L (0.0–0.80)	0.12 (0.05–0.28)
3-OH tetradecanoyl carnitine (C14-OH), μmol/L (0–0.12)	0.02 (0.00–0.07)
3-OH palmitoyl carnitine (C16-OH), μmol/L (0.0–0.1)	0.03 (0.00–0.10)
3-OH palmitoleyl carnitine (C16:1-OH), μmol/L (0.0–0.18)	0.02 (0.00–0.07)
3-OH oleyl carnitine (C18:1-OH), μmol/L (0.0–0.1)	0.01 (0.00–0.17)
3-OH linonenoyl carnitine (C18:2-OH), μmol/L (0.0–0.1)	0.01 (0.00–0.06)

**Table A2:** Comparison of plasma amino acid, carnitine, and acylcarnitine levels between the ICP-term and control groups.

Amino Acid, Units (Reference Values)	ICP-Term Group Mean (±SD)	Control Group Mean (±SD)	*p*-Value
Alanine, μmol/L (90–900)	211.00 (±72.00)	185.66(±47.62)	0.001
Methionine, μmol/L (9.0–65),	19.91 (±4.65)	19.31 (±3.95)	0.306
Phenylalanine, μmol/L (22.9–120)	49.07(±8.22)	48.73 (±9.02)	0.197
Tyrosine, μmol/L (26.9–275)	81.21 (±45.23)	71.95 (±46.05)	0.037
Leucine/Isoleucine (IIe), μmol/L (43.4–373)	116.96 (±34.20)	93.10 (±21.01)	0.001
Valine, μmol/L (52.8–250)	98.42 (±28.04)	90.97 (±23.14)	0.016
Arginine, μmol/L (0.0–50)	8.33 (±5.46)	7.23 (±4.53)	0.002
Citrulline, μmol/L (2.0–65)	10.32 (±3.87)	10.39 (±3.56)	0.717
Glycine, μmol/L (105–1100)	335.50 (±92.29)	292.47 (±88.13)	0.001
Ornithine, μmol/L (19.7–300)	74.97 (±37.78)	52.07 (±23.31)	0.001
Arginino-succinic acid μmol/L (0.0–0.66)	0.012 (±0.026)	0.019 (±0.039)	0.018
Glutamic acid, μmol/L (0.0–704)	250.91 (±62.09)	266.04 (±67.17)	0.017
**Acylcarnitines, units (reference values)**			
Free carnitine (C0), μmol/L (8.6–90.0)	22.16 (±7.51)	21.74 (±8.80)	0.779
**Short-Chain acylcarnitines**			
Acetyl carnitine (C2), μmol/L (5.0–73.4)	24.35 (±9.45)	24.22 (±8.90)	0.582
Propionyl carnitine (C3), μmol/L (0.0–6.8)	1.825 (±0.902)	2.273 (±1.009)	0.001
Butyryl carnitine (C4), μmol/L (0.0–1.2)	0.262 (±0.147)	0.247 (±0.178)	0.106
Isovaleryl carnitine (C5), μmol/L (0.0–0.6)	0.134 (±0.152)	0.105 (±0.045)	0.001
Tiglyl carnitine (C5:1), μmol/L (0.0–0.13)	0.014 (±0.008)	0.014 (±0.11)	0.880
**Medium-chain acylcarnitines**			
Hexonyl carnitine (C6), μmol/L (0.0–0.21)	0.053 (±0.52)	0.050 (±0.026)	0.718
Octanoyl carnitine (C8), μmol/L (0.0–0.32)	0.055 (±0.027)	0.055 (±0.041)	0.811
Decanoyl carnitine (C10), μmol/L (0.0–0.48)	0.076 (±0.033)	0.075 (±0.035)	0.794
Decenoyl carnitine (C10:1), μmol/L (0.0–0.28)	0.041 (±0.02)	0.032 (±0.01)	0.001
Dodecanoyl carnitine (C12), μmol/L (0.0–0.69)	0.094 (±0.061)	0.107 (±0.051)	0.029
**Long-Chain acylcarnitines**			
Tetradecanoyl carnitine (C14), μmol/L (0.0–0.8)	0.206 (±0.047)	0.215 (±0.082)	0.005
Tetradecenoyl carnitine (C14:1), μmol/L (0.0–0.6)	0.100 (±0.093)	0.127 (±0.077)	0.001
Tetradecadienoyl carnitine (C14:2), μmol/L (0.0–0.25)	0.027 (±0.014)	0.029 (±0.017)	0.416
Palmitoyl carnitine (C16), μmol/L (0.0–8.70)	2.813 (±1.042)	3.044 (±1.011)	0.034
Palmitoleyl carnitine (C16:1), μmol/L (0.0–1.04)	0.210 (±0.093)	0.254 (±0.081)	0.001
Stearyl carnitine (C18), μmol/L (0.0–2.24)	0.854 (±0.278)	0.851 (±0.267)	0.841
Oleyl carnitine (C18:1), μmol/L (0.0–2.8)	1.110 (±0.375)	0.831 (±0.316)	0.001
Linolenoyl carnitine (C18:2), μmol/L (0.0–0.9)	0.181 (±0.102)	0.150 (±0.080)	0.001
**Acylcarnitine esters derived from dicarboxylic acids-DC**			
Glutaryl carnitine (C5-DC), μmol/L (0.0–0.21)	0.067 (±0.037)	0.076 (±0.032)	0.006
Methylglutaryl carnitine (C6-DC), μmol/L (0.0–0.20)	0.026 (±0.015)	0.031 (±0.017)	0.001
**Acylcarnitine esters derived from hydroxylated acids-OH**			
3-OH butyryl carnitine (C4-OH), μmol/L (0.0–0.48)	0.148 (±0.087)	0.155 (±0.079)	0.942
3-OH isovaleryl carnitine (C5-OH), μmol/L (0.0–0.80)	0.120 (±0.037)	0.107 (±0.039)	0.008
3-OH tetradecanoyl carnitine (C14-OH), μmol/L (0–0.12)	0.025 (±0.011)	0.027 (±0.012)	0.053
3-OH palmitoyl carnitine (C16-OH), μmol/L (0.0–0.1)	0.033 (±0.016)	0.035 (±0.014)	0.316
3-OH palmitoleyl carnitine (C16:1-OH), μmol/L (0.0–0.18)	0.023 (±0.009)	0.026 (±0.011)	0.009
3-OH oleyl carnitine (C18:1-OH), μmol/L (0.0–0.1)	0.014 (±0.005)	0.015 (±0.010)	0.037
3-OH linonenoyl carnitine (C18:2-OH), μmol/L (0.0–0.1)	0.013 (±0.004)	0.012 (±0.007)	0.022
